# Supplementary material for: A nationwide questionnaire study of post-acute symptoms and health problems after SARS-CoV-2 infection in Denmark
Source: Nat Commun. 2022 Jul 21;13:4213. doi: 10.1038/s41467-022-31897-x (PMC9302226; doi:10.1038/s41467-022-31897-x)
Supplement: Supplementary file 1 — Supplementary Information [file 41467_2022_31897_MOESM1_ESM.pdf]

A nationwide Danish questionnaire study of post-acute symptoms and health problems after SARS-CoV-2 infection

Supplementary Information

Table of contents

Supplementary Fig. 1: Risk differences of symptoms around the test date, comparing SARS-CoV-2 test-positive and test-negative participants. 2

Supplementary Fig. 2: Risk differences of symptoms 6-12 months after test, comparing hospitalized and non-hospitalized SARS-CoV-2 test-positive participants. 3

Supplementary Fig. 3: Risk differences of self-reported health problems with new onset between the test date and until 6-12 months after, comparing SARS-CoV-2 test-positive and test-negative participants, stratified by sex and age group 4

Supplementary Fig. 4: Risk differences of self-reported diagnoses with new onset between the test date and until 6-12 months after, comparing SARS-CoV-2 test-positive and test-negative participants, stratified by sex and age group 5

Supplementary Table 1: Characteristics of participants, stratified by participation 6

Supplementary Table 2: Characteristics of participants, stratified by test status 8

Supplementary Table 3: Risk differences of post-acute symptoms reported after six, nine or twelve months, comparison of SARS-CoV-2 test-positive and test-negative participants 10

Supplementary Note 1: English translation of the questionnaire 11

Supplementary Fig. 1: Risk differences of symptoms around the test date, comparing SARS-CoV-2 test-positive and test-negative participants.

Symptom	Positive (n,%)	Negative (n,%)	Risk Difference (95% CI)
Dysgeusia	22,419 (36.8%)	237 (5.3%)	34.49 (33.74, 35.28)
Dysosmia	21,884 (35.9%)	247 (5.5%)	33.87 (33.06, 34.73)
Fever	33,577 (55.0%)	1,453 (32.5%)	23.90 (22.35, 25.28)
Chills	22,800 (37.4%)	810 (18.1%)	21.86 (20.59, 23.15)
Muscle/joint pain	25,835 (42.4%)	1,066 (23.9%)	21.54 (20.27, 22.99)
Reduced appetite	19,183 (31.4%)	639 (14.3%)	19.05 (17.94, 20.13)
Reduced strength legs/arms	15,770 (25.9%)	431 (9.7%)	17.33 (16.30, 18.28)
Hot flushes/sweat	21,220 (34.8%)	904 (20.2%)	16.37 (14.87, 17.73)
Fatigue/exhaustion	28,779 (47.2%)	1,515 (33.9%)	14.39 (12.85, 16.00)
Dizziness	13,440 (22.0%)	452 (10.1%)	13.77 (12.78, 14.73)
Dyspnea	12,213 (20.0%)	370 (8.3%)	13.60 (12.62, 14.44)
Headache	26,880 (44.1%)	1,481 (33.2%)	11.59 (10.00, 13.16)
Chest pain	9,003 (14.8%)	279 (6.2%)	10.01 (9.19, 10.73)
Nausea	7,450 (12.2%)	320 (7.2%)	6.41 (5.67, 7.25)
Sleeping legs/arms	5,524 (9.1%)	162 (3.6%)	6.19 (5.59, 6.75)
Diarrhoea	6,559 (10.8%)	301 (6.7%)	4.93 (4.17, 5.65)
Abdominal pain	5,103 (8.4%)	279 (6.2%)	3.17 (2.42, 3.87)
Red runny eyes	4,126 (6.8%)	208 (4.7%)	2.70 (2.01, 3.35)
Cough	21,917 (35.9%)	1,772 (39.7%)	−1.79 (−3.39, −0.27)
Runny nose	15,654 (25.7%)	1,770 (39.6%)	−11.98 (−13.58, −10.22)
Sore throat	17,902 (29.3%)	2,183 (48.9%)	−16.93 (−18.51, −15.27)

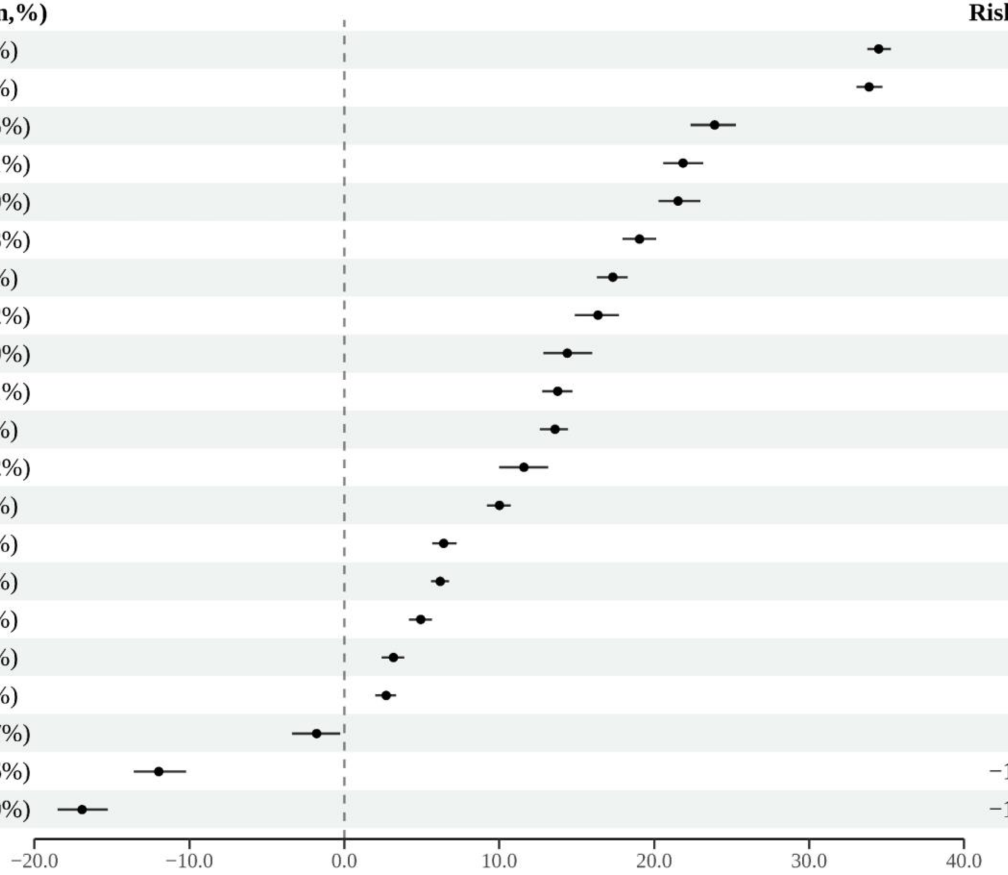

Note: Bars indicate risk differences between the test-positive and test-negative groups (centre) and 95% intervals (length of error bars) adjusted for age, sex, comorbidities, obesity, healthcare-occupation and time after testing (in months). “Around the test date” is defined as from one week prior to the test date and until four weeks after. For symptoms around the test date, test-negatives with symptoms as indication for testing are used as control population. All symptom questions were mandatory, so for all lines the proportions are based on 61,002 test-positive and 4.465 test-negative individuals. An individual could only participate in the study once, as either test-positive or test-negative.

Supplementary Fig. 2: Risk differences of symptoms 6-12 months after test, comparing hospitalized and non-hospitalized SARS-CoV-2 test-positive participants.

Symptom	Hospitalized (n,%)	Non−hospitalized (n,%)	Risk Difference (95% CI)
Fatigue/exhaustion	397 (16.4%)	6,402 (10.9%)	8.64 (6.70, 10.74)
Reduced strength legs/arms	302 (12.5%)	3,079 (5.3%)	7.13 (5.55, 8.66)
Dyspnea	274 (11.3%)	3,003 (5.1%)	6.71 (5.17, 8.39)
Muscle/joint pain	201 (8.3%)	3,016 (5.1%)	5.05 (3.57, 6.72)
Headache	180 (7.4%)	3,560 (6.1%)	4.58 (2.86, 6.31)
Sleeping legs/arms	203 (8.4%)	2,638 (4.5%)	4.25 (2.87, 5.68)
Dizziness	158 (6.5%)	2,272 (3.9%)	4.11 (2.64, 5.72)
Chest pain	121 (5.0%)	1,574 (2.7%)	4.02 (2.69, 5.38)
Cough	151 (6.2%)	2,805 (4.8%)	2.81 (1.45, 4.36)
Hot flushes/sweat	108 (4.5%)	1,939 (3.3%)	2.53 (1.34, 3.86)
Nausea	69 (2.9%)	1,110 (1.9%)	2.38 (1.24, 3.53)
Abdominal pain	71 (2.9%)	1,170 (2.0%)	1.88 (0.93, 2.87)
Reduced appetite	90 (3.7%)	1,682 (2.9%)	1.78 (0.63, 2.95)
Sore throat	95 (3.9%)	2,187 (3.7%)	1.52 (0.15, 2.80)
Fever	68 (2.8%)	1,294 (2.2%)	1.44 (0.43, 2.46)
Red runny eyes	58 (2.4%)	764 (1.3%)	1.41 (0.62, 2.32)
Chills	57 (2.4%)	909 (1.6%)	1.38 (0.53, 2.28)
Runny nose	92 (3.8%)	2,284 (3.9%)	1.15 (−0.12, 2.36)
Diarrhoea	57 (2.4%)	1,065 (1.8%)	0.98 (0.16, 1.79)
Dysgeusia	184 (7.6%)	5,181 (8.8%)	0.97 (−0.65, 2.69)
Dysosmia	188 (7.8%)	6,486 (11.1%)	−0.52 (−2.27, 1.21)

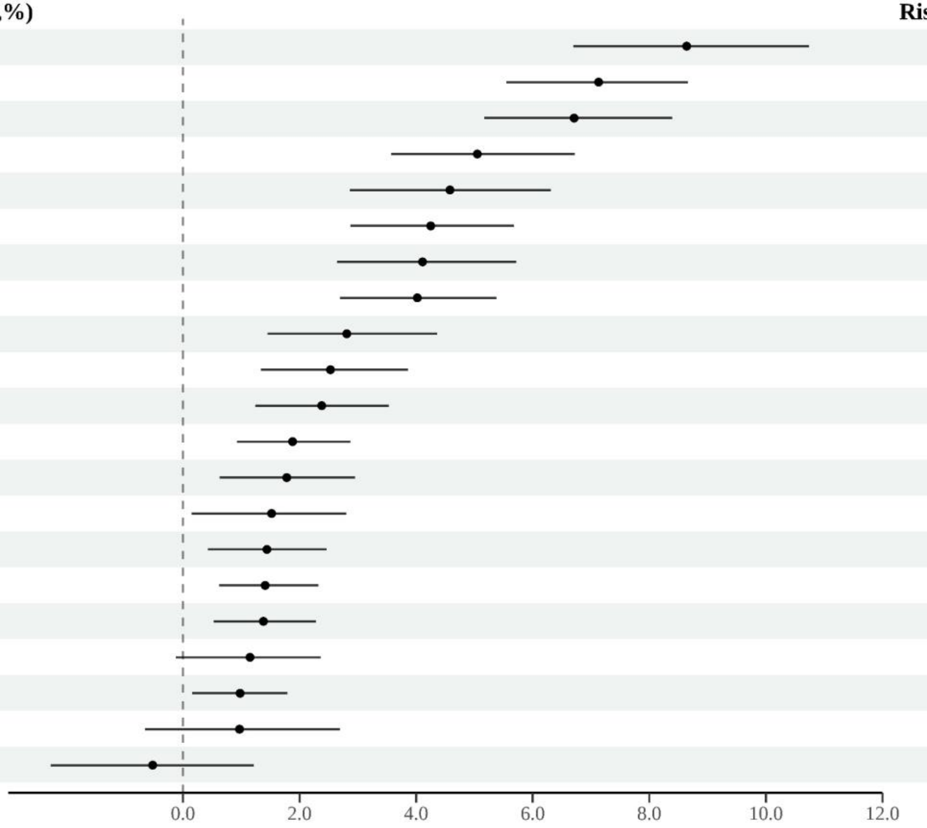

Note: Bars indicate risk differences (centre) between the hospitalized (n=2,421) and non-hospitalised groups (n=58,581) and 95% intervals (length of error bars) adjusted for age, sex, comorbidities, obesity, healthcare-occupation and time after testing (in months).

Supplementary Fig. 3: Risk differences of self-reported health problems with new onset between the test date and until 6-12 months after, comparing SARS-CoV-2 test-positive and test-negative participants, stratified by sex and age group

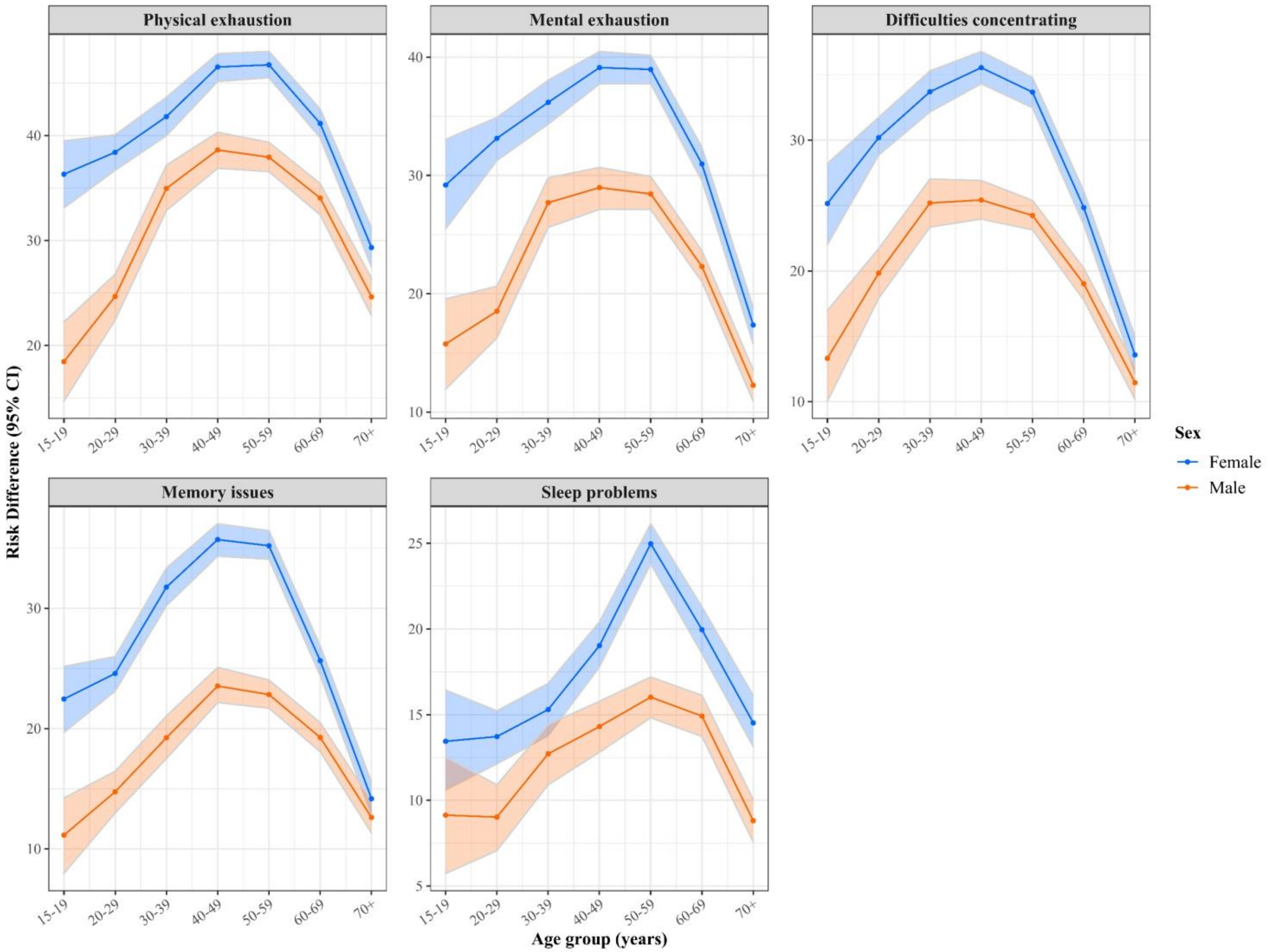

Note: Risk differences (centre) with 95% confidence intervals (length of error bands) were adjusted for comorbidities, obesity, healthcare-occupation and time after testing (in months).

Supplementary Fig. 4: Risk differences of self-reported diagnoses with new onset between the test date and until 6-12 months after, comparing SARS-CoV-2 test-positive and test-negative participants, stratified by sex and age group

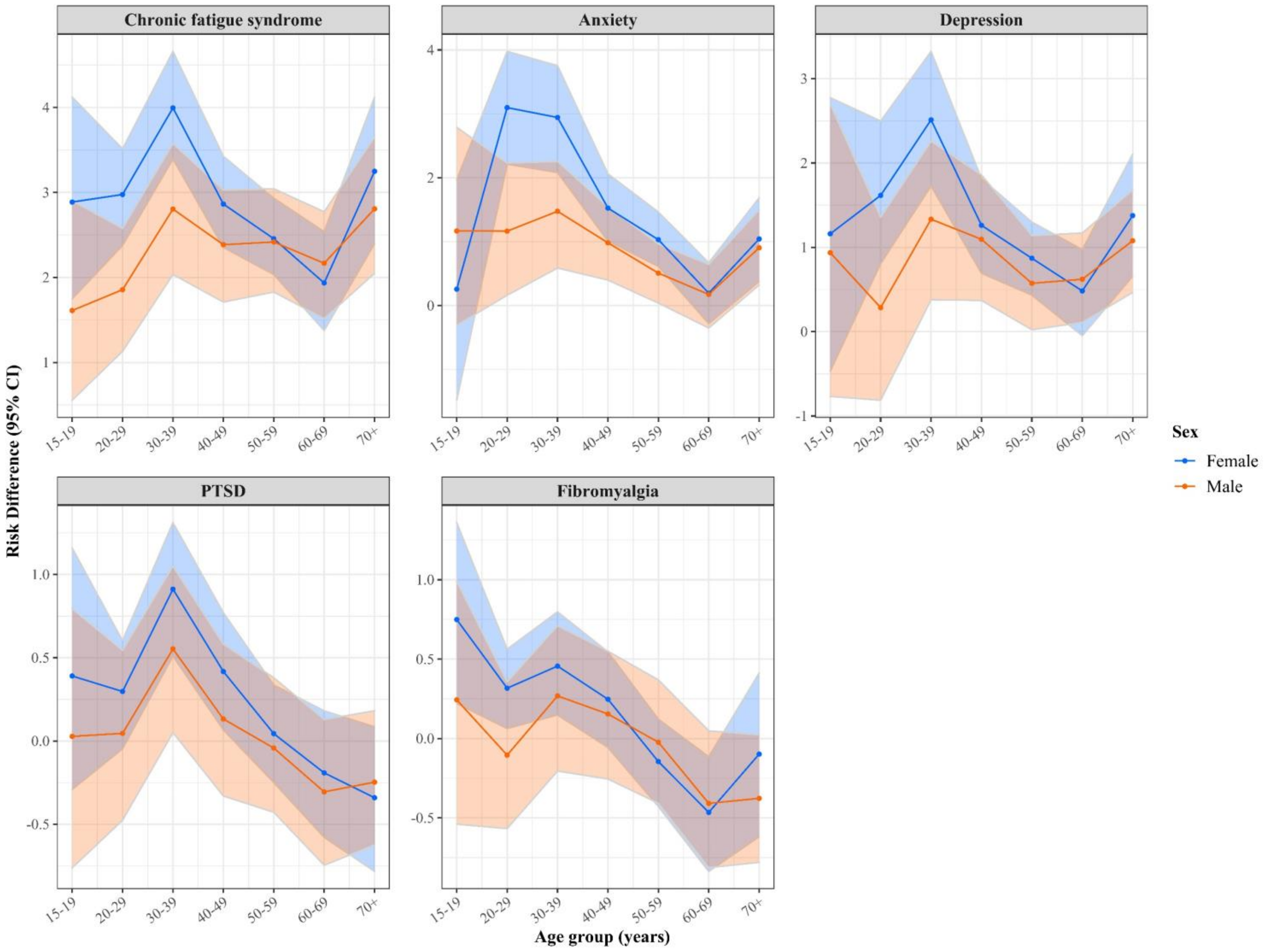

Note: Risk differences (centre) with 95% confidence intervals (width of error bands) were adjusted for comorbidities, obesity, healthcare-occupation and time after testing (in months).

Supplementary Table 1: Characteristics of participants, stratified by participation

	Non-participants	Full-participants	Partial-participants	p-value
**Test result (n,%)**				
Negative	154,085 (59.1%)	91,878 (60.1%)	11,687 (72.5%)	< 0.0001
Positive	106,552 (40.9%)	61,002 (39.9%)	4,438 (27.5%)	
**Sex (n, %)**				
Female	133,489 (51.2%)	93,494 (61.2%)	10,246 (63.5%)	< 0.0001
Male	127,148 (48.8%)	59,386 (38.8%)	5,879 (36.5%)	
**Age group (n,%)**				
15-19	33,372 (12.8%)	5,531 (3.6%)	1,757 (10.9%)	< 0.0001
20-29	68,006 (26.1%)	17,979 (11.8%)	3,025 (18.8%)	
30-39	50,752 (19.5%)	17,817 (11.7%)	2,621 (16.3%)	
40-49	47,302 (18.1%)	27,494 (18.0%)	2,825 (17.5%)	
50-59	35,868 (13.8%)	38,819 (25.4%)	2,866 (17.8%)	
60-69	15,522 (6.0%)	28,505 (18.6%)	1,694 (10.5%)	
70+	9,815 (3.8%)	16,735 (10.9%)	1,337 (8.3%)	
**Occupation (n,%)**				
Non-healthcare worker	239,173 (91.8%)	134,054 (87.7%)	14,551 (90.2%)	< 0.0001
Healthcare worker	21,464 (8.2%)	18,826 (12.3%)	1,574 (9.8%)	
**Elderly home (n,%)**				
No	258,830 (99.3%)	152,665 (99.9%)	16,060 (99.6%)	< 0.0001
Yes	1,807 (0.7%)	215 (0.1%)	65 (0.4%)	
**Origin (n,%)**				
Danish	198,669 (76.2%)	136,667 (89.4%)	13,097 (81.2%)	< 0.0001
Born abroad	47,158 (18.1%)	14,287 (9.3%)	2,536 (15.7%)	
Immigrant	14,797 (5.7%)	1,922 (1.3%)	490 (3.0%)	
**Region (n,%)**				
Hovedstaden	108,575 (41.7%)	56,010 (36.6%)	6,216 (38.5%)	< 0.0001
Midtjylland	50,097 (19.2%)	30,970 (20.3%)	3,293 (20.4%)	
Nordjylland	23,400 (9.0%)	15,210 (9.9%)	1,580 (9.8%)	
Sjælland	33,807 (13.0%)	21,911 (14.3%)	2,094 (13.0%)	
Syddanmark	44,522 (17.1%)	28,691 (18.8%)	2,933 (18.2%)	
NA	236 (0.1%)	88 (0.1%)	9 (0.1%)	
**Hospitalization due to COVID-19 n,%)**				
No	257,488 (98.8%)	150,379 (98.4%)	15,814 (98.1%)	< 0.0001
Yes	3,149 (1.2%)	2,501 (1.6%)	311 (1.9%)	

Note: All symptom questions were mandatory, so for all lines the proportions are based on 61,002 test-positive and 91,878 test-negative individuals. An individual could only participate in the study once, as either test-positive or test-negative.

P-values were estimated using student's t-test for continuous variables and Pearson's Chi-squared test for categorical variables. No adjustments for multiple comparisons were made. Detailed p-values: Test result: p = 3.22E-246; Sex: p <2e-16; Age group: p <2e-16; Occupation: p <2e-16; Elderly home: p <2e-16; Origin: p <2e-16; Region: p <2e-16 ; Hospitalization due to COVID-19: p <2e-16.

Charlson comorbidity scores were based on data from The Danish National Patient Register, whereas the listed comorbidities were self-reported.

Supplementary Table 2: Characteristics of participants, stratified by test status

	Positive	Negative	p-value
**Age group (n,%)**			
15-19	2,350 (3.9%)	3,181 (3.5%)	< 0.0001
20-29	9,021 (14.8%)	8,958 (9.7%)	
30-39	7,979 (13.1%)	9,838 (10.7%)	
40-49	11,377 (18.7%)	16,117 (17.5%)	
50-59	14,946 (24.5%)	23,873 (26.0%)	
60-69	9,530 (15.6%)	18,975 (20.7%)	
70+	5,799 (9.5%)	10,936 (11.9%)	
**Obesity (n,%)**			
Non-obese	46,256 (75.8%)	68,712 (74.8%)	< 0.0001
Obese	9,973 (16.3%)	15,073 (16.4%)	
No information	4,773 (7.8%)	8,093 (8.8%)	
**Charlson comorbidity score (n,%)**			
0	53,530 (87.8%)	78,694 (85.7%)	< 0.0001
1	3,807 (6.2%)	6,309 (6.9%)	
2 or more	3,665 (6.0%)	6,875 (7.4%)	
**Region (n, %)**			
Hovedstaden	26,242 (43.0%)	29,768 (32.4%)	< 0.0001
Midtjylland	11,233 (18.4%)	19,737 (21.5%)	
Syddanmark	9,557 (15.7%)	19,134 (20.8%)	
Sjælland	9,233 (15.1%)	12,678 (13.8%)	
Nordjylland	4,706 (7.7%)	10,504 (11.4%)	
No information	31 (0.1%)	57 (0.1%)	
**Origin (n, %)**			
Danish	52,622 (86.3%)	84,045 (91.5%)	< 0.0001
Born abroad	7,122 (11.7%)	7,165 (7.8%)	
Immigrant	1,257 (2.1%)	665 (0.7%)	
**Comorbidities (n, %)**			
Diabetes	2,647 (4.3%)	4,202 (4.6%)	0.03
Asthma	4,579 (7.5%)	6,167 (6.7%)	< 0.0001
Hypertension	8,842 (14.5%)	14,903 (16.2%)	< 0.0001
COPD or other chronic lung disease	1,187 (1.9%)	2,453 (2.7%)	< 0.0001
Chronic headache (including migraine)	2,138 (3.5%)	3,457 (3.8%)	0.01
Other chronic disease	7,968 (13.1%)	14,751 (16.1%)	< 0.0001
**Occupation (n, %)**			
Non-healthcare worker	54,400 (89.2%)	79,654 (86.7%)	< 0.0001
Healthcare worker	6,602 (10.8%)	12,224 (13.3%)	

Note: All symptom questions were mandatory, so for all lines the proportions are based on 61,002 test-positive and 91,878 test-negative individuals. An individual could only participate in the study once, as either test-positive or test-negative. COPD = Chronic Obstructive Pulmonary Disease.

P-values were estimated using student's t-test for continuous variables and Pearson's Chi-squared test for categorical variables**.** No adjustments for multiple comparisons were made. Detailed p-values: Age group: p<2e-16; Obesity: p = 4.75E-11; Charlson Comorbidity Score: p = 1.06E-33; Region: p<2e-16; Origin: 4.28E-266; Diabetes: p = 0.03111; Asthma: p = 2.89E-09; Hypertension: p = 7.81E-20; COPD or other chronic lung disease: p = 1.13E-19; Chronic headache (including migraine): p = 0.008927; Other chronic disease: p = 2.38E-58; Occupation: p = 2.35E-47.

Supplementary Table 3: Risk differences of post-acute symptoms reported after six, nine or twelve months, comparison of SARS-CoV-2 test-positive and test-negative participants

	6 months after test (n = 22 541)			9 months after test (n = 106 611)			12 months after test (n = 23 728)		
**Symptom**	**Positive (n,%)**	**Negative (n,%)**	**RD (95% CI)**	**Positive (n,%)**	**Negative (n,%)**	**RD (95% CI)**	**Positive (n,%)**	**Negative (n,%)**	**RD (95% CI)**
Dysosmia	702 (9.4%)	70 (0.5%)	9.38 (8.66, 10.20)	5,044 (11.5%)	422 (0.7%)	11.56 (11.24, 11.89)	928 (9.5%)	112 (0.8%)	9.26 (8.61, 9.92)
Dysgeusia	587 (7.8%)	59 (0.4%)	7.78 (7.13, 8.44)	4,008 (9.2%)	391 (0.6%)	9.06 (8.76, 9.36)	770 (7.9%)	101 (0.7%)	7.65 (7.05, 8.28)
Fatigue/exhaustion	920 (12.3%)	435 (2.9%)	9.79 (8.92, 10.66)	4,905 (11.2%)	1,959 (3.1%)	8.49 (8.14, 8.86)	974 (9.9%)	474 (3.4%)	6.95 (6.24, 7.77)
Dyspnea	450 (6.0%)	122 (0.8%)	5.70 (5.09, 6.29)	2,361 (5.4%)	556 (0.9%)	4.86 (4.61, 5.12)	466 (4.8%)	135 (1.0%)	4.19 (3.73, 4.69)
Reduced strength legs/arms	448 (6.0%)	140 (0.9%)	5.21 (4.57, 5.83)	2,448 (5.6%)	715 (1.1%)	4.71 (4.46, 5.00)	485 (5.0%)	169 (1.2%)	4.04 (3.53, 4.58)
Sleeping legs/arms	355 (4.7%)	174 (1.2%)	3.67 (3.12, 4.23)	2,053 (4.7%)	859 (1.4%)	3.53 (3.29, 3.78)	433 (4.4%)	203 (1.5%)	3.21 (2.68, 3.74)
Muscle/joint pain	429 (5.7%)	263 (1.7%)	4.05 (3.44, 4.73)	2,304 (5.3%)	1,236 (2.0%)	3.41 (3.15, 3.67)	484 (4.9%)	273 (2.0%)	3.17 (2.64, 3.75)
Headache	502 (6.7%)	413 (2.7%)	4.05 (3.36, 4.79)	2,657 (6.1%)	2,003 (3.2%)	2.93 (2.63, 3.25)	581 (5.9%)	452 (3.2%)	2.69 (2.11, 3.30)
Dizziness	322 (4.3%)	210 (1.4%)	2.86 (2.33, 3.45)	1,754 (4.0%)	1,049 (1.7%)	2.38 (2.15, 2.61)	354 (3.6%)	236 (1.7%)	1.91 (1.45, 2.42)
Chest pain	234 (3.1%)	90 (0.6%)	2.54 (2.13, 3.00)	1,195 (2.7%)	538 (0.9%)	1.97 (1.79, 2.15)	266 (2.7%)	152 (1.1%)	1.72 (1.34, 2.13)
Hot flushes/sweat	264 (3.5%)	225 (1.5%)	2.04 (1.52, 2.57)	1,474 (3.4%)	1,085 (1.7%)	1.63 (1.42, 1.85)	309 (3.2%)	240 (1.7%)	1.49 (1.03, 1.99)
Reduced appetite	242 (3.2%)	153 (1.0%)	2.08 (1.64, 2.54)	1,251 (2.9%)	815 (1.3%)	1.45 (1.26, 1.65)	279 (2.8%)	208 (1.5%)	1.32 (0.90, 1.75)
Chills	117 (1.6%)	134 (0.9%)	0.51 (0.18, 0.85)	702 (1.6%)	666 (1.1%)	0.48 (0.33, 0.63)	147 (1.5%)	180 (1.3%)	0.15 (-0.20, 0.49)
Red runny eyes	92 (1.2%)	97 (0.6%)	0.58 (0.30, 0.91)	583 (1.3%)	512 (0.8%)	0.48 (0.34, 0.61)	147 (1.5%)	139 (1.0%)	0.56 (0.26, 0.92)
Nausea	136 (1.8%)	166 (1.1%)	0.59 (0.22, 0.98)	846 (1.9%)	915 (1.5%)	0.41 (0.25, 0.59)	197 (2.0%)	213 (1.5%)	0.42 (0.04, 0.80)
Abdominal pain	156 (2.1%)	192 (1.3%)	0.83 (0.41, 1.25)	877 (2.0%)	1,005 (1.6%)	0.35 (0.18, 0.54)	208 (2.1%)	213 (1.5%)	0.51 (0.13, 0.89)
Diarrhoea	119 (1.6%)	190 (1.3%)	0.23 (-0.11, 0.57)	830 (1.9%)	962 (1.5%)	0.32 (0.14, 0.51)	173 (1.8%)	186 (1.3%)	0.53 (0.17, 0.88)
Fever	172 (2.3%)	206 (1.4%)	0.69 (0.28, 1.13)	978 (2.2%)	1,120 (1.8%)	0.27 (0.07, 0.47)	212 (2.2%)	258 (1.9%)	0.15 (-0.24, 0.56)
Cough	349 (4.6%)	592 (3.9%)	0.34 (-0.25, 0.96)	2,097 (4.8%)	2,789 (4.4%)	-0.02 (-0.29, 0.25)	510 (5.2%)	696 (5.0%)	-0.25 (-0.95, 0.36)
Runny nose	267 (3.6%)	527 (3.5%)	-0.26 (-0.80, 0.27)	1,710 (3.9%)	2,373 (3.8%)	-0.16 (-0.44, 0.09)	399 (4.1%)	574 (4.1%)	-0.41 (-0.96, 0.19)
Sore throat	276 (3.7%)	519 (3.5%)	-0.16 (-0.75, 0.45)	1,602 (3.7%)	2,583 (4.1%)	-0.79 (-1.04, -0.52)	404 (4.1%)	588 (4.2%)	-0.47 (-1.09, 0.17)

Supplementary Note 1: English translation of the questionnaire

Background information: This is a print of a web-based questionnaire containing activations. This means that when filling out the questionnaire electronically, it depends on test status and/or answers to previous questions, which questions a given participant will be asked next. In this print, activations will be indicated by explanatory text in square brackets. When send out to participants, their specific test date of reference was inserted in all places indicated by [*test date*]. In one some questions, the listed time intervals differed depended on whether participants received the questionnaire 6, 9 or 12 month after the test. In this example, the intervals for after 6 months are shown.

[Start page in the questionnaire]

Welcome to the EFTER-COVID baseline questionnaire

In the EFTER-COVID project, we are examining the health of the population during and after the coronavirus pandemic. **Your answers are important, regardless of whether you have tested positive or negative for coronavirus (COVID-19)**

Everyone invited to participate in the study has been randomly selected to receive a questionnaire with a particular focus on a certain area of their health. The questionnaire you have received focuses on physical symptoms.

Many thanks for your help.

[*Test-positive participants only*]:

These first questions are about how you have been since you tested positive at [*testdate*]

Which of the following conditions best describes how you were when you were at your <u>worst?</u>

Think about the period from a week before you tested positive until 4 weeks after.

(1) ☐ I had no symptoms

(2) ☐ I was ill at home

(3) ☐ I was hospitalised due to COVID-19

(4) ☐ I was hospitalised due to COVID-19 and was intubated with a ventilator

(5) ☐ I was hospitalised for reasons other than COVID-19

[*Test-positive participants only*]:

Did you take any of the following types of medication for your COVID-19 symptoms?

Think of the period from one week before you were tested at [*test date*] and until 4 weeks after.

(1) ☐ Painkillers/antipyretics

(2) ☐ Asthma medication

(3) ☐ Other - please specify _____

(4) ☐ I didn't take any medication

[*Test-negative participants only*]:

Why were you tested on the [*test date*]?

(1) □ I had symptoms

(2) □ I was a close contact of someone infected

(3) □ I suspected that I had been exposed to the infection

(4) □ Other

(5) □ I can't remember

Physical symptoms in the period around the test.

Have you at any point had any of the following symptoms? Please consider the period from the week leading up to your test on [*test date*] and until now.

Kindly mark all the symptoms you have had without considering the symptom cause. You can add further comments at the end of the questionnaire.

	Yes	No	Don't know
Fever	(1) □	(0) □	(99) □
Chills	(1) □	(0) □	(99) □
Hot flushes / sweating	(1) □	(0) □	(99) □
Headache	(1) □	(0) □	(99) □
Muscle or joint pain	(1) □	(0) □	(99) □
Fatigue or exhaustion	(1) □	(0) □	(99) □
Dizziness	(1) □	(0) □	(99) □
Red or watery eyes	(1) □	(0) □	(99) □
Runny or stuffy nose	(1) □	(0) □	(99) □

Continued from previous page: In the week leading up to the test on [*test date*], and until now, did you have any of the following symptoms?

Kindly mark all the symptoms you have had without considering the symptom cause. You can add further comments at the end of the questionnaire.

	Yes	No	Don't know
Reduced or altered sense of smell	(1) ☐	(0) ☐	(99) ☐
Reduced or altered sense of taste	(1) ☐	(0) ☐	(99) ☐
Cough	(1) ☐	(0) ☐	(99) ☐
Sore throat or pain when swallowing	(1) ☐	(0) ☐	(99) ☐
Shortness of breath	(1) ☐	(0) ☐	(99) ☐
Chest pain	(1) ☐	(0) ☐	(99) ☐
Nausea or vomiting	(1) ☐	(0) ☐	(99) ☐
Diarrhoea	(1) ☐	(0) ☐	(99) ☐
Abdominal pain	(1) ☐	(0) ☐	(99) ☐
Reduced or loss of appetite	(1) ☐	(0) ☐	(99) ☐
Reduced arm and leg strength	(1) ☐	(0) ☐	(99) ☐
Sleeping or tingling sensation, or other abnormal sensations in the legs and arms	(1) ☐	(0) ☐	(99) ☐
Other symptoms	(1) ☐	(0) ☐	(99) ☐

[*In this question, only lines related to symptoms, which the participants have replied "yes" to in the previous question are displayed*]

During which periods did you have symptoms?

"Around the test date" covers the period from a week before the test on [*test date*] and up to four weeks after.

Please mark all of the periods during which you have had the symptom. If you have trouble remembering, then please mark the periods to the best of your ability.

	Around the test date	1-2 months following the test	2-6 months following the test	The past 14 days
Fever	(1) ☐	(12) ☐	(26) ☐	(14) ☐
Chills	(1) ☐	(12) ☐	(26) ☐	(14) ☐
Hot flushes / sweating	(1) ☐	(12) ☐	(26) ☐	(14) ☐
Headache	(1) ☐	(12) ☐	(26) ☐	(14) ☐
Muscle or joint pain	(1) ☐	(12) ☐	(26) ☐	(14) ☐
Fatigue or exhaustion	(1) ☐	(12) ☐	(26) ☐	(14) ☐
Dizziness	(1) ☐	(12) ☐	(26) ☐	(14) ☐
Red or watery eyes	(1) ☐	(12) ☐	(26) ☐	(14) ☐
Runny or stuffy nose	(1) ☐	(12) ☐	(26) ☐	(14) ☐
Reduced or altered sense of smell	(1) ☐	(12) ☐	(26) ☐	(14) ☐

Reduced or altered sense of taste	(1) ☐	(12) ☐	(26) ☐	(14) ☐
Sore throat or pain when swallowing	(1) ☐	(12) ☐	(26) ☐	(14) ☐
Cough	(1) ☐	(12) ☐	(26) ☐	(14) ☐
Shortness of breath	(1) ☐	(12) ☐	(26) ☐	(14) ☐
Chest pain	(1) ☐	(12) ☐	(26) ☐	(14) ☐
Nausea or vomiting	(1) ☐	(12) ☐	(26) ☐	(14) ☐
Diarrhoea	(1) ☐	(12) ☐	(26) ☐	(14) ☐
Abdominal pain	(1) ☐	(12) ☐	(26) ☐	(14) ☐
Reduced or loss of appetite	(1) ☐	(12) ☐	(26) ☐	(14) ☐
Reduced arm and leg strength	(1) ☐	(12) ☐	(26) ☐	(14) ☐
Sleeping or tingling sensation, or other abnormal sensations in the legs and arms	(1) ☐	(12) ☐	(26) ☐	(14) ☐

[*Only participants, who replied "yes" to having had fever*]:

Did you take your temperature when you had a fever in the period around your test on the [*test date*]?

(1) ☐ Yes (please write the highest temperature taken in the box below) ______

(0) ☐ No

(99) ☐ Don't know/Can't remember

[*Only participants, who replied "yes" to having had fever*]:

Were there known/probable causes other than COVID-19 for you having a fever in the period around the test on [*test date*]?

(1) ☐ Yes (please explain in the box below) ______

(0) ☐ No

(99) ☐ Don't know

[*In this question, only lines related to symptoms, which the participants have replied "yes" to having experienced, will be displayed*]

Before the test on [*test date*], did you generally (i.e. often or chronically) suffer from any of the following physical symptoms?

	Yes	No	Don't know
Fever	(1) ☐	(0) ☐	(99) ☐
Chills	(1) ☐	(0) ☐	(99) ☐
Hot flushes / sweating	(1) ☐	(0) ☐	(99) ☐
Headache	(1) ☐	(0) ☐	(99) ☐
Muscle or joint pain	(1) ☐	(0) ☐	(99) ☐
Fatigue or exhaustion	(1) ☐	(0) ☐	(99) ☐
Dizziness	(1) ☐	(0) ☐	(99) ☐
Red or watery eyes	(1) ☐	(0) ☐	(99) ☐

Runny or stuffy nose	(1) □	(0) □	(99) □
Reduced or altered sense of smell	(1) □	(0) □	(99) □
Reduced or altered sense of taste	(1) □	(0) □	(99) □
Sore throat or pain when swallowing	(1) □	(0) □	(99) □
Cough	(1) □	(0) □	(99) □
Shortness of breath	(1) □	(0) □	(99) □
Chest pain	(1) □	(0) □	(99) □
Nausea or vomiting	(1) □	(0) □	(99) □
Diarrhoea	(1) □	(0) □	(99) □
Abdominal pain	(1) □	(0) □	(99) □
Reduced or loss of appetite	(1) □	(0) □	(99) □
Reduced arm and leg strength	(1) □	(0) □	(99) □
Sleeping or tingling sensation, or other abnormal sensations in the legs and arms	(1) □	(0) □	(99) □

[*Only participants, who replied "yes" to having had headache. Only lines representing time intervals, where participants indicated having headache are displayed*]:

How severe were your headaches when you were at your worst during the specified period(s)?

	No pain	Mild pain	Moderate pain	Strong pain	Unbearable pain
Generally before the test date	(1) ☐	(2) ☐	(3) ☐	(4) ☐	(5) ☐
Around the test date	(1) ☐	(2) ☐	(3) ☐	(4) ☐	(5) ☐
1-2 months following the test	(1) ☐	(2) ☐	(3) ☐	(4) ☐	(5) ☐
2-6 months following the test	(1) ☐	(2) ☐	(3) ☐	(4) ☐	(5) ☐
The past 14 days	(1) ☐	(2) ☐	(3) ☐	(4) ☐	(5) ☐

[*Only participants, who replied "yes" to having had muscle or joint pain. Only lines representing time intervals, where participants indicated having this are displayed*]:

How bad was your muscle or joint pain in the specified period(s)?

	No pain	Mild pain	Moderate pain	Strong pain	Unbearable pain
Generally before the test date	(1) ☐	(2) ☐	(3) ☐	(4) ☐	(5) ☐
Around the test date	(1) ☐	(2) ☐	(3) ☐	(4) ☐	(5) ☐
1-2 months following the test	(1) ☐	(2) ☐	(3) ☐	(4) ☐	(5) ☐
2-6 months following the test	(1) ☐	(2) ☐	(3) ☐	(4) ☐	(5) ☐
The past 14 days	(1) ☐	(2) ☐	(3) ☐	(4) ☐	(5) ☐

[*Only participants, who replied "yes" to having had reduced or changed sense of smell. Only lines representing time intervals, where participants indicated having this are displayed*]:

How was your sense of smell in the specified period(s), compared to normal?

My sense of smell was/is…

	Slightly reduced	Very reduced	Completely gone	Changed	Don't know
Generally before the test date	(1) ☐	(2) ☐	(3) ☐	(4) ☐	(5) ☐
Around the test date	(1) ☐	(2) ☐	(3) ☐	(4) ☐	(5) ☐
1-2 months following the test	(1) ☐	(2) ☐	(3) ☐	(4) ☐	(5) ☐
2-6 months following the test	(1) ☐	(2) ☐	(3) ☐	(4) ☐	(5) ☐
The past 14 days	(1) ☐	(2) ☐	(3) ☐	(4) ☐	(5) ☐

[*Only participants, who replied "yes" to having had reduced or changed sense of taste. Only lines representing time intervals, where participants indicated having this are displayed*]:

How was your sense of taste in the specified period(s), compared to normal?

My sense of taste was/is…

	Slightly reduced	Very reduced	Completely gone	Changed	Don't know
Generally before the test date	(1) ☐	(2) ☐	(3) ☐	(4) ☐	(5) ☐
Around the test date	(1) ☐	(2) ☐	(3) ☐	(4) ☐	(5) ☐
1-2 months following the test	(1) ☐	(2) ☐	(3) ☐	(4) ☐	(5) ☐

2-6 months following the test	(1) ☐	(2) ☐	(3) ☐	(4) ☐	(5) ☐
The past 14 days	(1) ☐	(2) ☐	(3) ☐	(4) ☐	(5) ☐

[*Only participants, who replied "yes" to having experienced shortness of breath. Only lines representing time intervals, where participants indicated having this are displayed*]:

Choose the following statement below that best describes your shortness of breath during the relevant period(s).

A. I only become short of breath when I exert myself a lot

B. I become short of breath when I hurry or walk up a small hill

C. I walk slower than others my age due to my shortness of breath, or I have to stop to catch my breath when walking on even terrain

D. I stop to catch my breath after about 100 metres or a few minutes of walking

E. I have too much shortness of breath to leave my home, or I get short of breath when getting (un)dressed

	A	B	C	D	E	Don't know
Generally before the test date	(1) ☐	(2) ☐	(3) ☐	(4) ☐	(5) ☐	(99) ☐
Around the test date	(1) ☐	(2) ☐	(3) ☐	(4) ☐	(5) ☐	(99) ☐
1-2 months following the test	(1) ☐	(2) ☐	(3) ☐	(4) ☐	(5) ☐	(99) ☐
2-6 months following the test	(1) ☐	(2) ☐	(3) ☐	(4) ☐	(5) ☐	(99) ☐
In the past 14 days	(1) ☐	(2) ☐	(3) ☐	(4) ☐	(5) ☐	(99) ☐

[Only participants indicating having had other symptoms]:

Which other symptoms have you had and when?

____
____
____
____
____
____

[Only participants indicating having had other symptoms]:

Did you also used to have these symptoms generally (i.e. chronically or often) before the test on [*test date*]?

____
____
____
____
____
____

[*test-positives only*]:

Did your health return to normal within 4 weeks of testing positive for COVID-19?

(0) ☐ No, my health at this time was not yet completely back to as it was before I became ill

(1) ☐ Yes, my health returned to normal a number of days after symptoms started (write the number of days in the field below) _____

(99) ☐ Don't know

Did you take sick leave around the time of the test on [*test date*], or any time since then?

Please mark all of the relevant answers, regardless of the cause of illness

(1) ☐ Yes, I was on sick leave in the week up to the test or up to 4 weeks after

(2) ☐ Yes, I was on sick leave more than 4 weeks after the test

(0) ☐ No

(3) ☐ Not relevant

[*Only shown for participants, who indicated taking sick leave around the test date*]:

How long were you on sick leave in the period around the test?

Think about the period from one week before the test on [test date] and up to 4 weeks after.

Please write the number of sick days in the box below. If you can't remember the exact number, write an approximate amount of days. Have you not been on sick leave, please write '0' in the boxes below.

Full-time sick leave:

_____

Part-time sick leave:

_____

[*Only shown for participants, who indicated taking sick leave later than 4 weeks after the test date*]:

If you were on sick leave later than 4 weeks after the test date, were you on full-time or part-time sick leave?

If you have been on both full-time and part-time sick leave, please mark both answers below

(1) ☐ Full-time sick leave

(2) ☐ Part-time sick leave

[*test-positives only*]:

Was your sick leave related to you having had COVID-19?

(1) ☐ Yes

(0) ☐ No

(2) ☐ Partly

(99) ☐ Don't know

[*Only participants, who indicated having been on full-time sick leave*]:

How long were you on full-time sick leave?

(2) ☐ Less than 2 weeks

(4) ☐ 2-4 weeks

(12) ☐ 1-2 months

(24) ☐ 2-4 months

(46) ☐ 4-6 months

(10,000) ☐ I have been on full-time sick leave since the test date

[*Only participants, who indicated having been on part-time sick leave*]:

How long were you on part-time sick leave?

(2) Less than 2 weeks

(4) 2-4 weeks

(12) 1-2 months

(24) 2-4 months

(46) 4-6 months

(10,000) I have been on part-time sick leave since the test date

[*Only participants, who replied indicated having been on sick leave*]:

Has any of your sick leave been within the past 14 days?

(1) Yes

(0) No

(3) Not relevant

The following pages contain some background questions about your health, education, work and lifestyle.

Please provide your height in cm

If you don't know your height, or do not wish to answer, please click "Next" below.

_____

Please provide your approximate weight in kg.

If you don’t know your weight, or do not wish to answer, please click “Next” below

_____

What is your highest level of education?

(1) Primary/Elementary school (9th–10th grade)

(2) General secondary education or vocational secondary education

(3) Vocational training

(4) Shorter term higher education (1–2 years), e.g. vocational academy

(5) Medium term higher education (2–4 years), e.g. nursing, primary school teaching, BSc

(6) Longer term higher education (more than 5 years), e.g. Masters or PhD

(99) Don’t know/None of the above/Do not wish to answer

What is your main occupational status?

(1) Employed full-time

(2) Employed part-time

(3) Self-employed

(4) Student

(5) Stay-at-home parent or on parental leave

(6) Jobseeking/Unemployed

(7) Benefits recipient

(8) Long-term sick leave

(9) Pensioner or early retiree

(10) Other

Do you smoke or have you previously smoked?

(1) I've never smoked

(2) It has been more than 5 years since I smoked

(3) I have smoked within the past 5 years, but no longer smokes

(4) I smoke occasionally, e.g. social events

(5) I smoke daily (fewer than 10 cigarettes/cigars/pipes per day)

(6) I smoke daily (10 or more cigarettes/cigars/pipes per day)

(7) I smoke e-cigarettes/I vape

Please indicate how many alcoholic drinks you have on average per week.

One drink is for example 1 beer (330ml), 1 glass of wine (120ml), 1 glass of fortified wine (80ml), or 1 shot of spirits (40ml).

If you don't know, or don't wish to answer, please click "Next" below.

_____

If you look at the six months before the test on [*test date*], what would you say best describes your level of physical activity in your spare time?

(1) Train hard and play competitive sports regularly, several times a week

(2) Work out or do heavy gardening or similar at least 4 times a week

(3) Walk, cycle or other light exercise at least 4 times a week (including leisure walks, light gardening, and cycling/walking to work)

(4) Read, watch TV or other sedentary lifestyle

If you look at the six months before the test on [*test date*], how would you describe your physical fitness?

(1) ☐ Really good

(2) ☐ Good

(3) ☐ Fair

(4) ☐ Less good

(5) ☐ Poor

In the period six months before the test on [*test date*] and up to now, have you had any of the following problems?

Please mark all the relevant answers

	Yes, before the test date	Yes, after the test date	No	Don't know
Difficulties concentrating	(1) ☐	(2) ☐	(0) ☐	(99) ☐
Issues with memory	(1) ☐	(2) ☐	(0) ☐	(99) ☐
Mental exhaustion	(1) ☐	(2) ☐	(0) ☐	(99) ☐
Physical exhaustion	(1) ☐	(2) ☐	(0) ☐	(99) ☐
Sleep problems	(1) ☐	(2) ☐	(0) ☐	(99) ☐

Have you ever been diagnosed by a doctor with any of the following conditions?

	Yes, before the test date	Yes, after the test date	No
Depression	(1) ☐	(2) ☐	(0) ☐
Anxiety	(1) ☐	(2) ☐	(0) ☐
PTSD (Post traumatic stress disorder)	(1) ☐	(2) ☐	(0) ☐
Chronic fatigue syndrome	(1) ☐	(2) ☐	(0) ☐
Fibromyalgia	(1) ☐	(2) ☐	(0) ☐

Before the test on [*test date*], were you diagnosed with any chronic disease(s)?

Please mark all relevant.

(1) ☐ No known chronic diseases

(2) ☐ Diabetes

(3) ☐ Asthma

(4) ☐ High blood pressure

(5) ☐ COPD or other chronic lung disease

(6) ☐ Chronic or frequent headaches, including migraines

(7) ☐ Other chronic diseases

(8) ☐ Do not wish to respond

[*Test-negatives only*]:

Do you think that you have ever had covid-19?

(1) ☐ Yes

(0) ☐ No

(99) ☐ Don't know

[*Test-negatives, replying yes to the previous question*]:

Why do you think you probably had covid-19?

Please mark all relevant.

(1) ☐ Antibodies were detected in my blood sample

(2) ☐ I had symptoms and I think it was covid-19

(3) ☐ I had symptoms, and talked to a doctor who thought it was covid-19

(4) ☐ For other reasons

Many thanks for taking the time to complete our questionnaire.

May we contact you again?

(1) ☐ Yes

(0) ☐ No

If you have any further comments, feel free to write them in the box below.

Please, remember to click “Finish”

____

____

____

____

____

____
