# Supplementary material for: A nationwide questionnaire study of post-acute symptoms and health problems after SARS-CoV-2 infection in Denmark
Source: Nat Commun. 2022 Jul 21;13:4213. doi: 10.1038/s41467-022-31897-x (PMC9302226; doi:10.1038/s41467-022-31897-x)
Supplement: Supplementary file 3 — Description of Additional Supplementary Files [file 41467_2022_31897_MOESM3_ESM.pdf]

Description of Additional Supplementary Files

File Name: Supplementary Data 1
Description: Risk differences of symptoms after 6-12 months, comparing SARS-CoV-2 test-positive and test-negative participants, stratified by sex and age group

File Name: Supplementary Data 2
Description: Risk differences of self-reported health problems with new onset between the test date and until 6-12 months after, comparing SARS-CoV-2 test-positive and test-negative participants, stratified by sex and age group

File Name: Supplementary Data 3
Description: Risk differences of self-reported diagnoses with new onset between the test date and until 6-12 months, after comparing SARS-CoV-2 test-positive and test-negative participants, stratified by sex and age group
